# Research on a machine vision-based electro-killing pheromone-baited intelligent agricultural pest monitoring method

**DOI:** 10.3389/fpls.2025.1521594

**Published:** 2025-03-06

**Authors:** Guozhi Li, Zhengbo Liu, Zelin Feng, Jun Lyu, Bin Li, Guo Chen, Qing Yao

**Affiliations:** ^1^ School of Information Science and Engineering, Zhejiang Sci-Tech University, Hangzhou, China; ^2^ School of Electronic Science and Engineering, University of Electronic Science and Technology of China, Chengdu, China; ^3^ Guizhou Aerospace intelligent Agriculture Co., Ltd, China Aerospace Science and Industry Corporation, Guiyang, China; ^4^ School of Information and Control, Keyi College of Zhejiang Sci-Tech University, Shaoxing, China; ^5^ School of Computer Science and Technology (School of Artificial Intelligence), Zhejiang Sci-Tech University, Hangzhou, China

**Keywords:** agricultural pests, machine vision, smart electro-killing pheromone traps, YOLOv9-TrapPest, pest pheromone monitoring platform

## Abstract

The annual global economic losses from pest-induced crop damage are substantial and difficult to quantify. Real-time monitoring of pest dynamics and timely control strategies are crucial for food security. Among the primary monitoring techniques, sex pheromone-baited trapping technology is instrumental in the detection and management of agricultural pests. To address existing limitations-such as manual insect collection and counting in conventional traps, inaccuracies in photoelectric counting devices, and the requirement for manual replacement of sticky boards in image-based traps—an advanced agricultural pest monitoring system utilizing sex pheromone bait was designed and developed in this paper. The system integrates smart electro-killing pheromone traps, a pest detection model, and a pheromone monitoring platform. Male pests attracted to pheromones are neutralized by an electric grid and deposited on an image acquisition platform. A network camera captures images of the pests, which are processed by a YOLOv9-TrapPest detection model to identify and quantify them. This model incorporates an AKConv module to enhance feature extraction, reducing false detections from limb separation. The CBAM-PANet structure improves detection rates of sticky pests, while the FocalNet module optimizes fine-grained feature capture, excluding non-target pests. The YOLOv9-TrapPest model outperforms other detection models, achieving 97.5% average precision and 98.3% mAP50 for detecting seven pest species. Furthermore, a pest pheromone monitoring platform displays the images and identification results, supporting pest control decisions. This system incorporates automated functions for pest trapping, killing, counting, and clearing, thereby achieving complete automation in the monitoring of pests attracted by sex pheromones.

## 1 Introduction

The annual global economic losses caused by pests damaging crops are incalculable (Savary et al., 2019). Real-time monitoring of pest population dynamics, combined with the timely implementation of effective control measures, is crucial for ensuring food security. In the realm of agricultural pest monitoring, alongside traditional manual field survey methods, various intelligent devices are frequently employed. These include ground light traps, sex pheromone-baited traps, searchlight traps, and insect radar, which are utilized to trap and monitor pests. The trapped pests are subsequently identified and counted, enabling accurate detection and reporting of pest populations (Jiang et al., 2021). Among them, sex pheromone-baited traps, which utilize synthetic pheromone lures to trap target pest males, are commonly used for the physical control and monitoring of pests. They have been widely used in agricultural pest monitoring and control due to their simplicity, low cost, and environmental friendliness.

At present, sex pheromone-baited traps can be classified into two main categories based on the methods used for capturing and counting pests attracted by pheromone lures. These categories have been widely adopted in the field for pest monitoring and management. (1) Simple sex pheromone-baited traps are traps that utilize sticky boards or buckets to capture pests and employ a manual identification and counting method (Yang et al., 2020). (2) Bucket traps are traps designed to prevent pests from escaping and use photoelectric counters to automatically count the pests (Potamitis et al., 2014; Zhou, 2020). The two types of traps have the following shortcomings: (1) Simple sex pheromone-baited traps, though cost-effective, require regular field visits by surveyors to check the number of trapped insects and manually report the data. This process is both time-consuming and labor-intensive. Moreover, the data are not available in real-time and are difficult to track for historical analysis. Additionally, these traps are easily damaged and cannot be reused. (2) Traps utilizing sticky boards necessitate timely manual replacement of the boards when large numbers of insects are captured, further increasing labor demands. (3) Since synthetic pheromone lures cannot guarantee a high degree of specificity, and the proportion of pheromone lures’ components may vary across regions for a particular pest, these lures often attract various similar pest species (Shen et al., 2019), or non-target pests that mistakenly enter the trap. This leads to inflated counts of target pests by photoelectric counters and thus reduces the reliability of target pest monitoring (Luo et al., 2016).

With the continuous development of machine vision technology and its successful application across various domains, a growing number of researchers are exploring its integration with sticky boards in pest sex pheromone-baited monitoring systems to improve the intelligence and precision of pest monitoring. In this approach, pests are first attracted by pheromone lures, and they are glued to death when they touch the sticky boards. The machine vision system then captures images of the sticky boards, which are subsequently analyzed using pattern recognition or deep learning techniques to identify and count the trapped pests. Traditional pattern recognition methods typically follow these steps: background segmentation, feature extraction of the pests, and the identification and counting of target pests using similarity measures or classifiers (Yalcin, 2015; Li et al., 2019). Due to prolonged exposure of sticky boards to the environment, the image backgrounds can become complex. When a large number of pests are captured, challenges such as pests sticking together and interference from visually similar species arise. Traditional pattern recognition methods often struggle to handle these complexities, leading to poor generalization and a reduced ability to continuously and accurately identify pests. Consequently, these methods have not seen widespread adoption in the field. In recent years, the exceptional performance of deep learning in object recognition and target detection has led to its application in pheromone-baited pests images recognition. For instance, Ding and Taylor (2016) utilized a sliding window approach and convolutional neural network to identify codling moths on pheromone-baited sticky board images. Qiu et al. (2021) used the YOLOv5 model to identify *Spodoptera frugiperda* on both the collecting boards of the pest image monitoring device FAAS-Pest Monitor, and the sticky boards, achieving a precision rate of 96.23% and a recall rate of 91.85%. However, the study was limited to a single pest species, restricting its broader applicability to other pest types in agricultural monitoring systems. Tian et al. (2023) designed a pest monitoring device to capture pest images, and proposed the MD-YOLO model to detect three major orchard pests *Adoxophyes orana*, *Grapholitha molesta Busck*, and *Carposina niponensis Walsingham*, on sticky board images. The model achieved a mAP50 of 86.2%. Zhang et al. (2022) designed smart sex pheromone-baited traps to capture images of *Cnaphalocrocis medinalis* on sticky boards. To address the reduced recognition rate caused by complex backgrounds, sticky pests, and false detections of similar pests, a two-layer network model incorporating an improved YOLOv3 and DBTNet-101 was developed. This model achieved a recognition precision of 97.6% and a recall rate of 98.6%. However, the image capture modules of these sex pheromone-baited traps are not enclosed. The sticky boards are exposed to the environment and the pests on the boards can damaged and decayed, which cause missed and false detections of model. Moreover, the background complexity of the sticky boards images increases over time, making the pest identification more difficult.

To address the challenges of frequent replacements of sticky boards and the increasing complexity of image backgrounds due to outdoor exposure, an electro-killing intelligent monitoring system was developed for agricultural pests based on machine vision. In this system, to eliminate the need of frequent replacements of sticky boards, we replace sticky boards with an electro-killing mechanism and utilize a rotating flat bottom tray to clear the pests. And the electro-killing mechanism is enclosed, which prevents the image background from becoming more complex, thereby reducing the missed and false detections of model. Moreover, to address specific challenges in pest recognition for images captured by electro-killing mechanisms, such as false detections of pests with fragmented bodies and similar pest species, missed detections of pests adhere to one another, we designed the YOLOv9-TrapPest pest identification model by incorporating the AKConv module, CBAM-PANet structure, and FocalNet module into the YOLOv9 model. This system realizes the unattended sex pheromone-baited pest monitoring, including automatic pest attraction, electrocution of pests, image capture, pest identification, pest counting, and data traceability. And it provides real-time data support for the monitoring and control of agricultural pests attracted by pheromone lures.

## 2 Materials and methods

### 2.1 Overall system architecture

**Figure 1:**
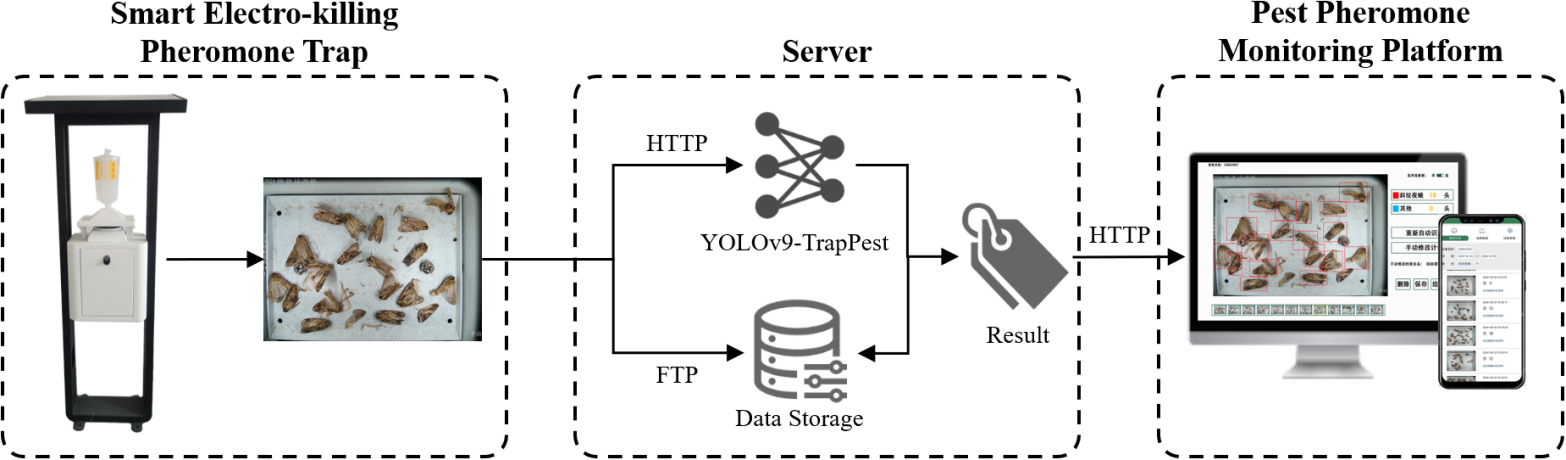
Architecture of the electro-killing pheromone-baited intelligent agricultural pest monitoring system.

The electro-killing pheromone-baited intelligent agricultural pest monitoring system mainly consists of three modules, smart electrical pheromone traps, a server for data access and model deployment, and a platform for pest reporting, as shown in **Figure 1**. The smart electrical pheromone trap is responsible for trapping and electrocuting the pests and capturing the pest images. These images are then transferred to the server via FTP protocol, where a pest identification and counting model is deployed. The results are subsequently displayed on the platform.

### 2.2 Smart electro-killing pheromone trap

**Figure 2:**
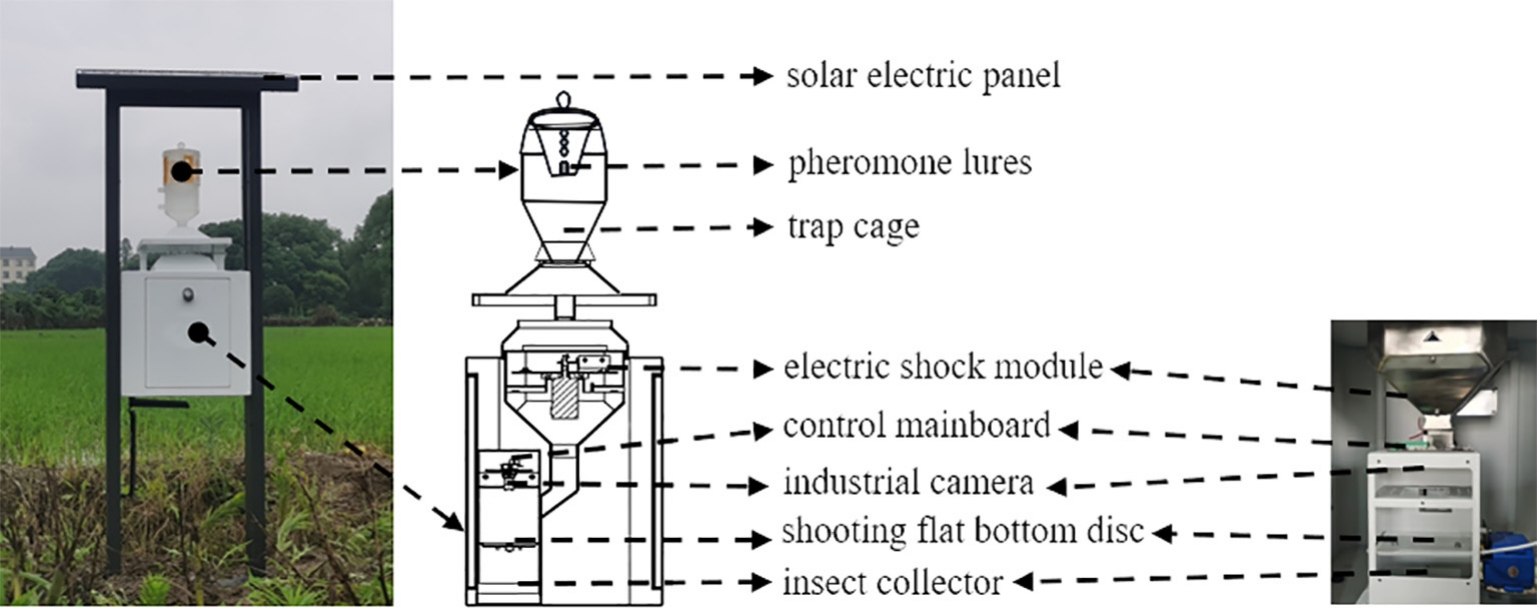
The smart electrical pheromone trap.

The smart electro-killing pheromone trap is mainly composed of four parts: solar electric panel, trap cage, electric shock module and machine vision module. The structure is shown in **Figure 2**. Among them, the machine vision module includes a control mainboard, a 1200w pixel industrial camera, a shooting flat bottom tray, a flip motor, and an insect collector.

Given the challenges of obtaining a reliable electricity supply in remote farmland, solar panels are utilized as the power source for the smart electro-killing pheromone traps, ensuring environmental sustainability. A dedicated software system has been developed on the control mainboard to manage various functions. This includes controlling the timing of the power supply to the electric grid, activating the camera to capture images, and rotating the flat bottom tray 360°, facilitating comprehensive pest monitoring and system efficiency.

Smart electro-killing pheromone traps attract pests using pheromone lures placed within the trap cage. When a pest enters the trap, it collides with the cage wall or cover and falls onto the electric shock module, where it is electrocuted. Since a large number of pests may lead to pest adhesion, we will set the number of times the electric grid operates to ensure that the number of trapped pests does not become too large. The pest then falls through the gaps in the electric grid of the shock module onto the flat bottom tray of the machine vision module. The control mainboard schedules the camera to automatically capture images of the pests on the flat bottom tray. The image files are uploaded to the server via FTP protocol for storage, while additional data—such as device number, device status, GIS coordinates, fault information, and image paths—are transmitted via HTTP protocol and stored in the server’s database. The server processes the uploaded images using an automatic identification and counting model to perform data analysis and generate statistical reports. Afterward, the control mainboard activates a flip motor that rotates the flat bottom tray 360° to reset it, causing the pests to fall into an insect collector located at the bottom of the trap. This automated sequence allows the smart electro-killing pheromone trap to fully automate the entire process of pest attraction, electrocution, image capture, data transmission, and analysis. As a result, the system enables completely unattended operation for pheromone-baited pest monitoring.

### 2.3 Image dataset

From 2023 to 2024, a total of 7 smart electro-killing pheromone traps were deployed across farms located in Jinhua City, Zhejiang Province; Zhuhai City, Guangdong Province; and Ningjiang District, Songyuan City, Jilin Province. Each device was equipped with a specific pheromone lure for monitoring pheromone-baited pests. Over the course of the deployment, 1,796 images were collected, capturing seven types of agricultural pests. The images, each with a resolution of 4000×3000 pixels, were stored in JPG format. The detailed data information is presented in **Table 1**.

**Table 1:** Information on seven agricultural pests.

Pest species	Images	Number of images	Number of targets	Number of targets after data enhancement
*Chilo suppressalis*	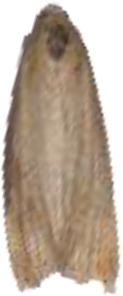	379	2341	18727
*Cnaphalocrocis medinalis*	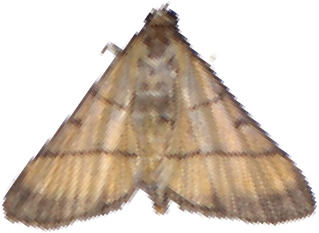	298	2415	19320
*Spodoptera frugiperda*	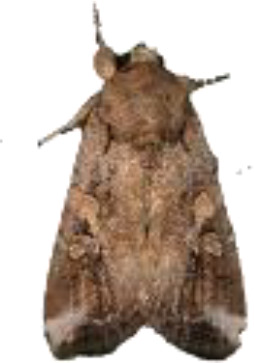	201	1357	10856
*Sesamia inferens*	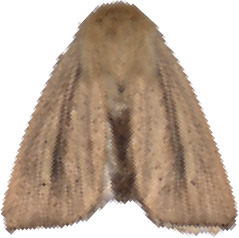	235	1221	9768
*Leucania loreyi*	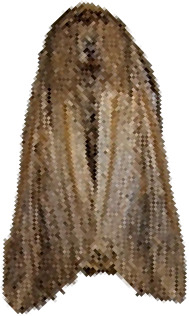	214	1643	13144
*Pyrausta nubilalis*	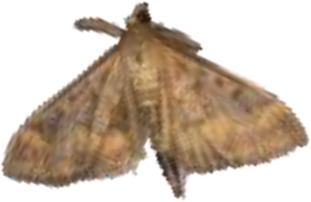	223	2013	16104
*Spodoptera litura*	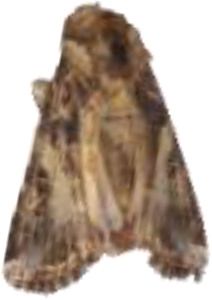	246	1545	12360

The LabelImg tool was used to annotate the pests in the images, with category information and location information saved in XML files. After labelling, the pest image data was converted into YOLO dataset format. However, due to the insufficient solar power supply caused by consecutive rainy and cloudy days, the voltage provided by the solar panels was low. This low voltage led to images with insufficient brightness. To address this issue, the brightness augmentation technique was applied to enhance image quality, ensuring that the final detection results were not affected. Further, to improve the generalization capabilities of the pest detection model, additional data augmentation techniques were applied. These include rotation, mirroring, noise addition, and mosaic augmentation, helping the model become more robust in recognizing pests under various environmental and image conditions. The dataset was then split into training sets, validation sets and test sets in an 8:1:1 ratio.

### 2.4 YOLOv9-TrapPest model

YOLOv9 (Wang et al., 2024) is an improved target detection model built upon the YOLO framework. It utilizes the Programmable Gradient Information (PGI) technique to generate more reliable gradients through reversible branches, thereby improving the convergence and performance of the model. Additionally, the model introduces the Generalized Efficient Layer Aggregation Network (GELAN) to optimize parameter utilization and computational efficiency. Experimental results showed that YOLOv9 achieved performance comparable to YOLOv7AF (Wang et al., 2023) on the MS COCO dataset (Lin et al., 2014) with an AP of 53%, while using 42% fewer parameters and 21% less computational power. This makes it particularly suitable for resource-constrained environments. To meet the requirements for high-precision pest identification and counting in real-time monitoring systems while maintaining a lightweight and efficient model, the YOLOv9-TrapPest model was developed in this paper, based on YOLOv9, to detect and count seven types of agricultural pests in the images captured by smart electrical pheromone traps.

**Figure 3:**
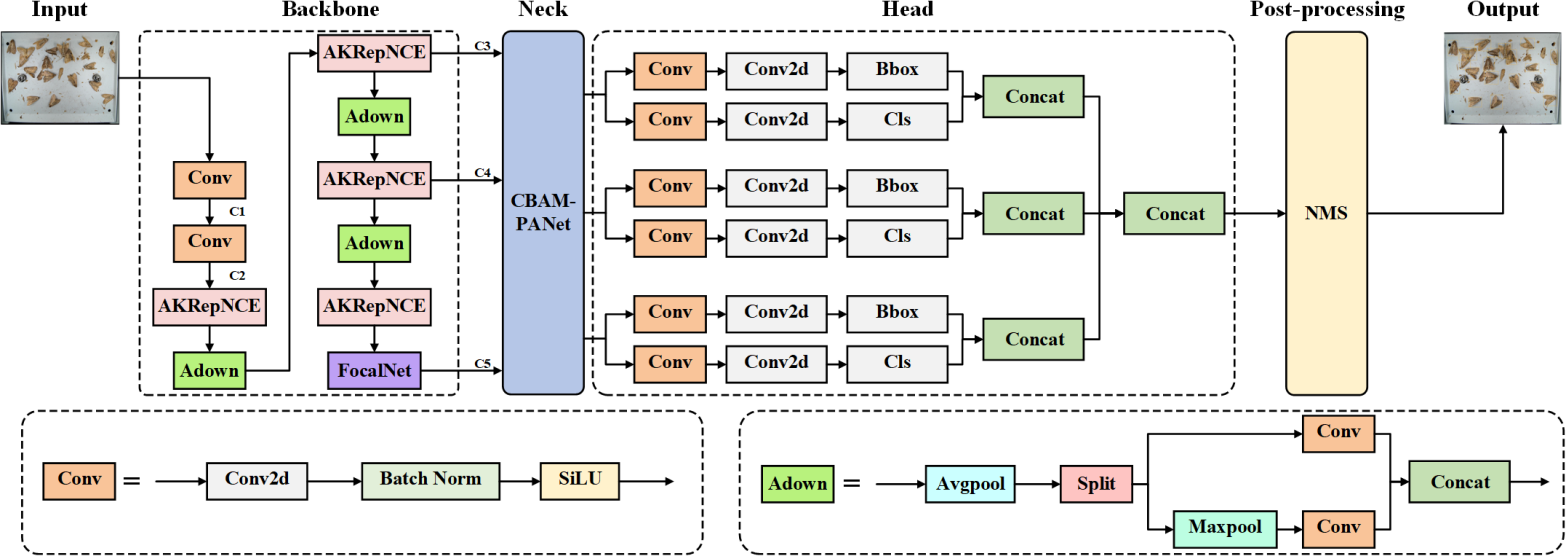
The structure of the YOLOv9-TrapPest model.

The difficulty of model identification is increased by the fact that pests struggle when they touch the electric grid after being lured over by the pheromone lures, which tends to cause separation or fragmentation of the pest limbs. In this paper, Alterable Kernel Convolution (AKConv) (Zhang et al., 2023) was introduced into the backbone network to improve the feature extraction and fusion module, RepNCSPELAN4, to reduce the interference of pests’ limbs. During peak pest periods, heavy adhesion of pests leads to a reduction in the model’s recall rate. To address this issue, we proposed the CBAM-PANet structure based on the attention mechanism, which improved the Path Aggregation Network (PANet) structure (Liu et al., 2018) in the Neck network of YOLOv9. By incorporating both channel attention and spatial attention mechanisms, the model’s ability to focus on important features and critical regions was significantly enhanced (Woo et al., 2018), allowing for more accurate identification and localization of targets in images with complex backgrounds and adhered pests. Although pheromone lures are specific, some closely related species have similar chemical components in their pheromone lures, which can easily attract similar pests. To reduce the false detections of similar pests, the Focal Modulation Network (FocalNet) (Yang et al., 2022) was employed in this paper to replace the SPPELAN module in the Head network. By introducing the focal modulation mechanism, the feature extraction network’s capacity to focus on high-confidence regions was enhanced, allowing the model to capture fine-grained features more effectively. This improvement enables the model to better distinguish between similar pest species by concentrating on the most relevant areas within an image. The structure of the YOLOv9-TrapPest model is illustrated in **Figure 3**.

#### 2.4.1 AKRepNCE

RepNCSPELAN4 is the feature extraction-fusion module in YOLOv9, which combines two architectures with gradient path planning: CSPNet and ELAN. It is designed as a generalized efficient layer aggregation network, considering factors such as lightweight design, inference speed, and accuracy.

**Figure 4:**
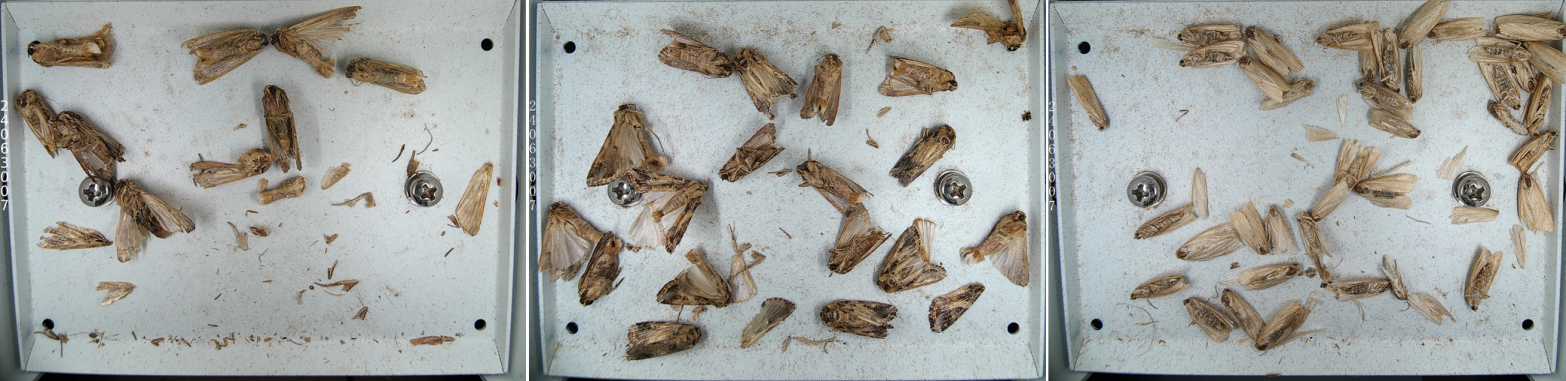
Selected images of agricultural pests.

**Figure 5:**
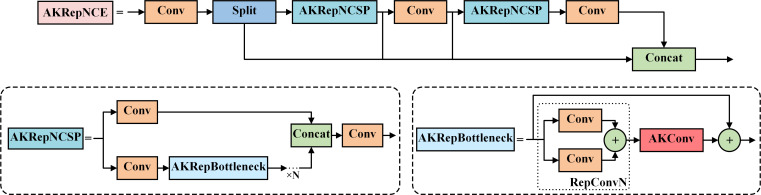
The structure of the AKRepNCE.

In convolutional neural networks, standard convolution operations are limited to a local window of fixed shape, with the kernel size fixed at *k × k*, resulting in a quadratic growth in the number of parameters. In pest recognition, the fixed sampling windows and convolutional kernels make it difficult for the model to accurately identify pests with partial or damaged bodies caused by the electric shock module, which in turn reduces the model’s recognition accuracy, as shown in **Figure 4**. Therefore, in this paper, the AKConv was introduced to enhance the feature extraction and fusion capabilities of the RepNCSPELAN4 module. The improved module, named AKRepNCE, and its model structure is shown in **Figure 5**.

AKConv enables the convolution kernel to adaptively adjust its shape and parameters according to the features of the input image by integrating Deformable Convolution and Dynamic Convolution techniques. This flexibility enables the kernel to better accommodate pests with different morphologies. Even when the body parts of pests are disarticulated or fragmented, the convolutional kernel can adaptively identify the primary characteristics of the pest, without being influenced by the disjointed fragments.

#### 2.4.2 CBAM-PANet

**Figure 6:**
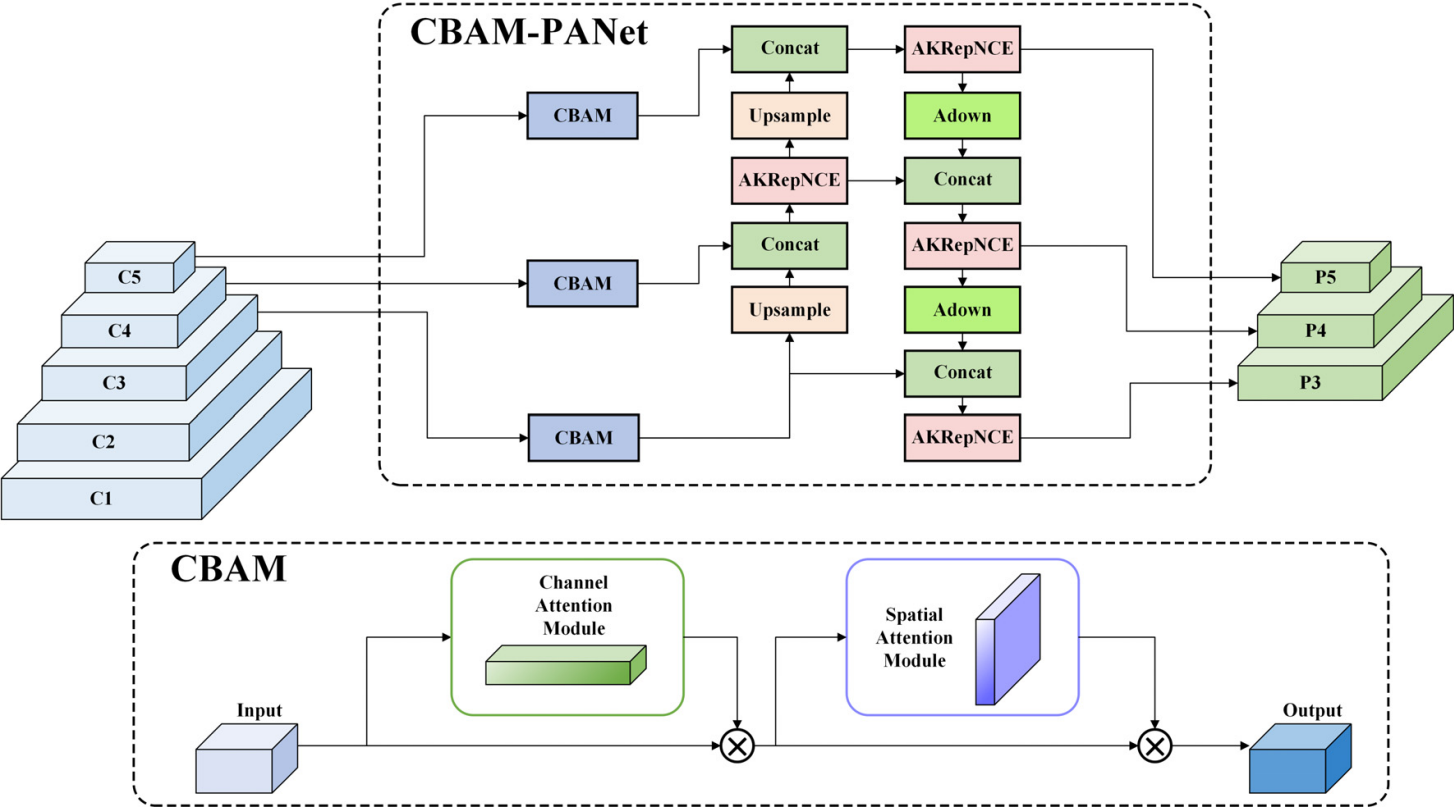
The structure of the CBAM-PANet.

The neck network of YOLOv9 adopts PANet, which introduces a bottom-up path augmentation structure based on the Feature Pyramid Network (Lin et al., 2017). PANet improves the locational expressiveness of the feature pyramid by introducing the bottom-up path aggregation mechanism, enabling the upward transfer and fusion of low-level feature information. This enhancement notably strengthens the model’s ability to accurately detect objects across varying scales and in complex backgrounds. However, during peak periods of agricultural pest infestations, pests may adhere to one another, leading to issues of missed detection. To address this, the CBAM attention mechanism was integrated into the PANet architecture, resulting in the development of the CBAM-PANet module. The CBAM attention mechanism combines channel attention and spatial attention, enhancing the model’s focus on key features. As shown in **Figure 6**, regarding the input feature map, the CBAM attention mechanism infers attention maps sequentially across two independent dimensions—channel and spatial. These maps are then multiplied with the input feature map to adaptively refine features, resulting in enhanced feature details. By emphasizing critical features in both the channel and spatial dimensions, the model can more accurately identify and localize target pests that are adhered together.

#### 2.4.3 FocalNet

YOLOv9 employs the SPPELAN module for spatial pyramid pooling, which integrates spatial pyramid pooling with an enhanced local attention network. However, in the context of this paper, it demonstrates some limitations in effectively handling feature diversity and multi-scale features. To more accurately identify similar agricultural pests (for example, as show in **Figure 4**, *Spodoptera frugiperda* and *Spodoptera litura* in this paper, which are difficult to recognize because many of the insects are positioned belly-up), the SPPELAN module was replaced with FocalNet.

**Figure 7:**
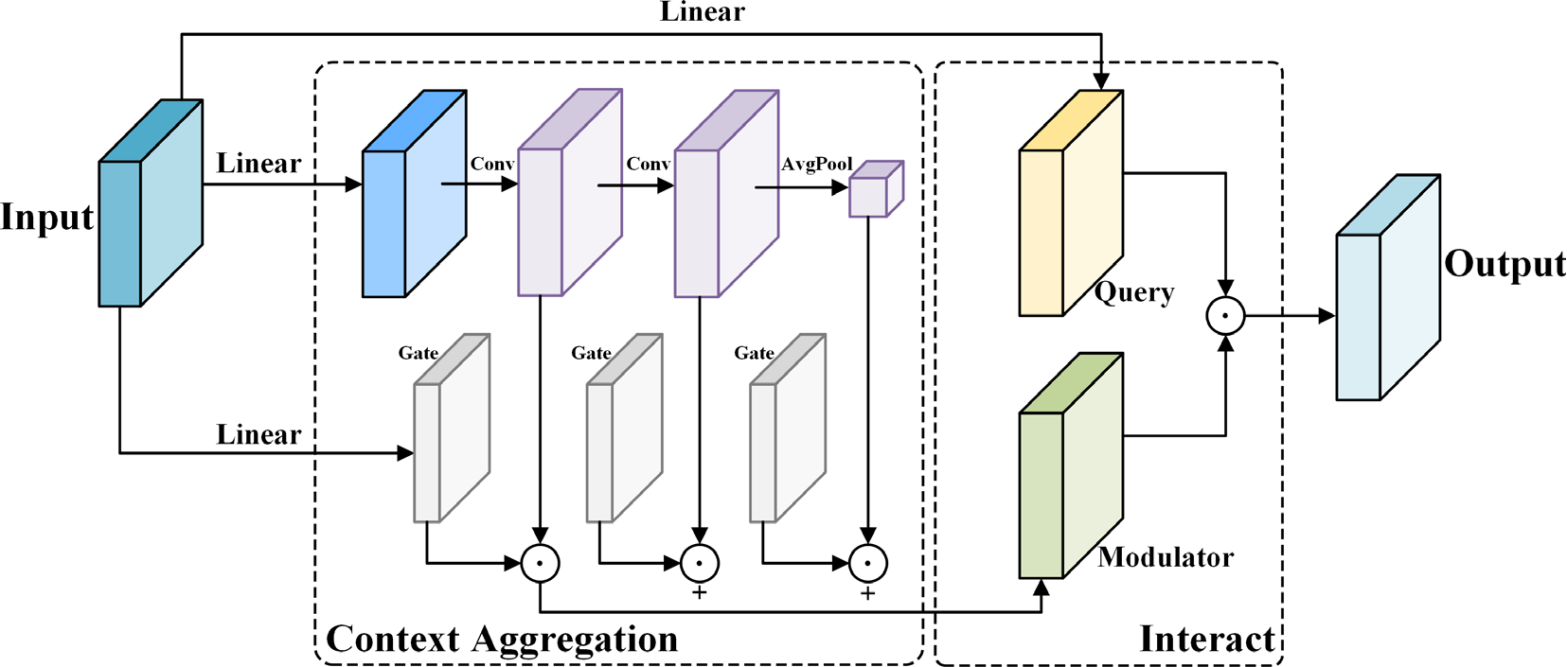
The structure of the Focal Modulation Network module.

In computer vision, different regions of an image contribute variably to a given task. Some regions contain critical information necessary for classification or detection, while others consist of background or irrelevant elements. The core concept of FocalNet is to replace the self-attention module with a focal modulation mechanism, which captures long-range dependencies and contextual information within the image. This mechanism allows the model to concentrate more precisely on essential features while reducing focus on irrelevant areas, enabling a more refined and targeted modulation of features. This approach not only improves the model’s sensitivity to key features but also reduces the computational complexity typically associated with traditional self-attention mechanisms. The network architecture of FocalNet is depicted in **Figure 7**.

FocalNet obtains contextual and global information by projecting input feature vectors into a new feature space and then using deep convolution and global average pooling. These features are then compressed into modulators and a dot product operation is performed using gated weights *G* to obtain the intermediate output *Z^out^* as shown in (Equation 1).


(1) $$\begin{array}{c} Z^{out}=\sum_{l=1}^{L+1}G^{l}⊙Z^{l} \end{array}$$

*Z^l^* represents the feature representation of layer *l* and *G^l^* is the corresponding gating weight. Finally, the final output *y_i_* is obtained by combining the linear layer *h* and the query *q* to achieve the communication between different channels, as shown in (Equation 2).


(2) $$\begin{array}{c} y_{i}=q(x_{i})⊙h(\sum_{l=1}^{L+1}{ɡ}_{i}^{l}\cdot z_{i}^{l}) \end{array}$$

With the integration of FocalNet, the model can effectively minimize background noise interference and more accurately capture small yet important feature differences within an image. This capability is especially beneficial for agricultural pest detection tasks, where it allows for the differentiation between pest species with visually similar characteristics, thereby reducing the likelihood of false detections.

### 2.5 Experimental configuration

The computer processor used in this study is an Intel Core i7 9700, with a base clock frequency of 3.0 GHz. The system is equipped with an 8TB hard drive and 16GB of memory. The graphics card is an RTX 1080 Ti. The development environment includes Ubuntu 20.04, Python 3.6, Anaconda 9.12, and CUDA version 11.6. During model training, the following hyperparameters were configured: the number of epochs was set to 200, the momentum was set to 0.937, the weight decay was set to 0.0005, and the batch size was set to 4.

### 2.6 Performance evaluation

To objectively evaluate the detection effect of the proposed agricultural pest detection model, Precision (P), Recall (R), Average Precision (AP), and mean Average Precision (mAP) were selected as the evaluation metrics, and the formulas were computed as shown in (Equations 3–6), respectively.

Precision *P_k_* indicates the proportion of correctly identified target pests of class *k* in all samples identified as that target pest of class *k*. Recall *R_k_* indicates the proportion of correctly identified target pests of class *k* in all samples of that target pest of class *k*. The average precision *AP_k_* integrates precision and recall, and the model is evaluated by calculating the area under the precision-recall curves at different thresholds to assess the model performance. *mAP* is the mean value of the average precision of all pest classes, which is a key indicator for comprehensively evaluating the model performance in multi-classification tasks. A higher *mAP* value indicates better detection performance, as it reflects the model’s ability to accurately detect and classify multiple pest species.


(3) $$\begin{array}{c} P_{k}=\frac{TP_{k}}{TP_{k}+FP_{k}} \end{array}$$

(4) $$\begin{array}{c} R_{k}=\frac{TP_{k}}{TP_{k}+FN_{k}} \end{array}$$

(5) $$\begin{array}{c} AP_{k}=\int_{0}^{1}P_{k}(R_{k})dR_{k} \end{array}$$

(6) $$\begin{array}{c} mAP=\frac{\sum_{k=1}^{n}AP_{k}}{n} \end{array}$$

### 2.7 Pest pheromone monitoring platform

The pest pheromone monitoring platform adopts the architecture of front-end and back-end separation. The front-end includes the Web end and App end. The interactive interface for the Web end was developed using Vue, while the App end was built on Android. The back-end utilizes the SpringBoot framework to handle data processing and other operations, with MySQL serving as the database for data storage.

**Figure 8:**
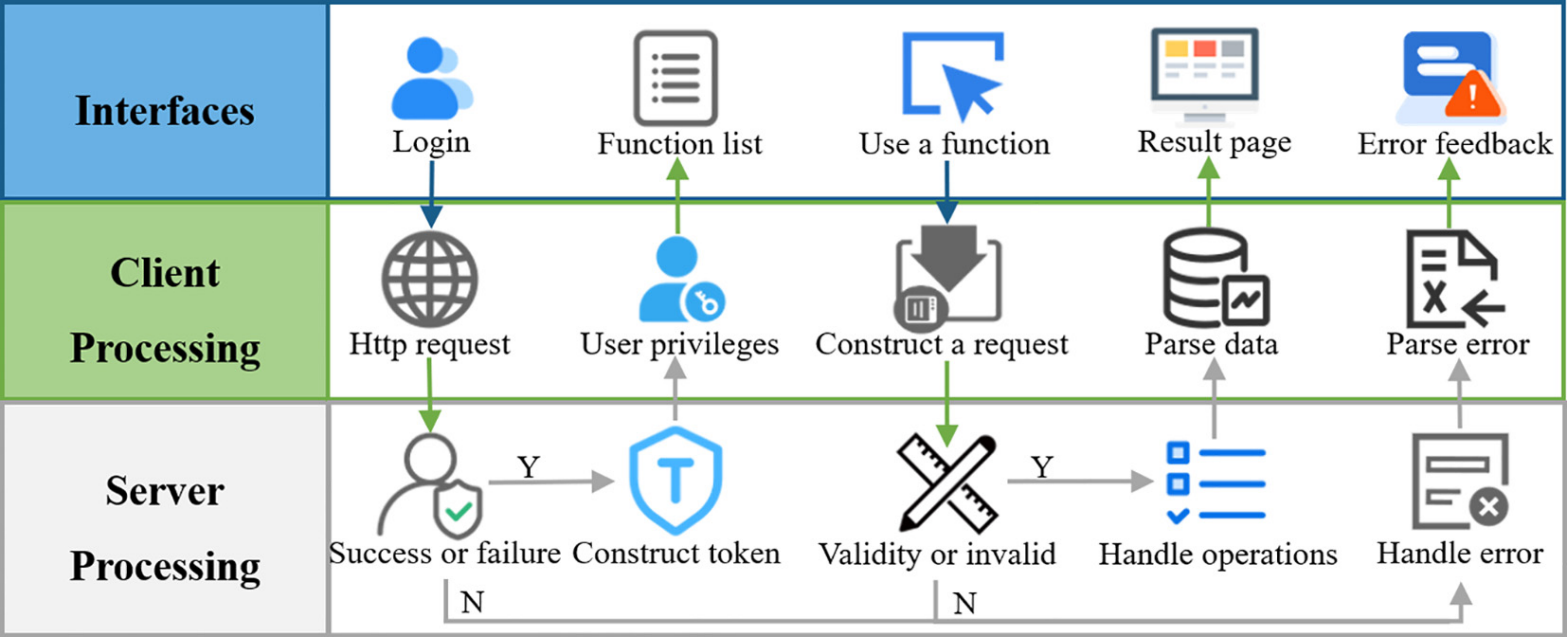
The pest pheromone monitoring platform.

The functions of the pest pheromone monitoring platform are shown in **Figure 8**, and include features such as an image list, data analysis, and device management. After logging in, users can access the image list interface to view detailed information about pest identification results. Through the data analysis interface, users can review pest statistics, while the device management interface allows them to remotely send control commands. These commands can be used to configure equipment operating modes, set image capture times, and manually control the equipment to take pictures. The system ensures seamless collaboration between the front and back ends, providing users with accurate, real-time pest monitoring and analysis data.

## 3 Results

### 3.1 Ablation experiment

To validate the effectiveness of the three improved strategies in the proposed YOLOv9-TrapPest model for pest recognition, the Precision, Recall, and mAP50 of each improved model were compared using the same test set. The results of the ablation experiments are summarized in **Table 2**, where the mark “✓” indicates the improvement strategy applied.

**Table 2:** Impact of three improved strategies on pest identification based on YOLOv9.

Exp	AKConv	CBAM-PANet	FocalNet	Recall (%)	Precision (%)	mAP50 (%)	
1				91.5	92.2	93.9
2	✓			93.4	91.6	95.1
3	✓	✓		93.1	95.2	97.2
4	✓	✓	✓	96.6	97.5	98.3

**Figure 9:**
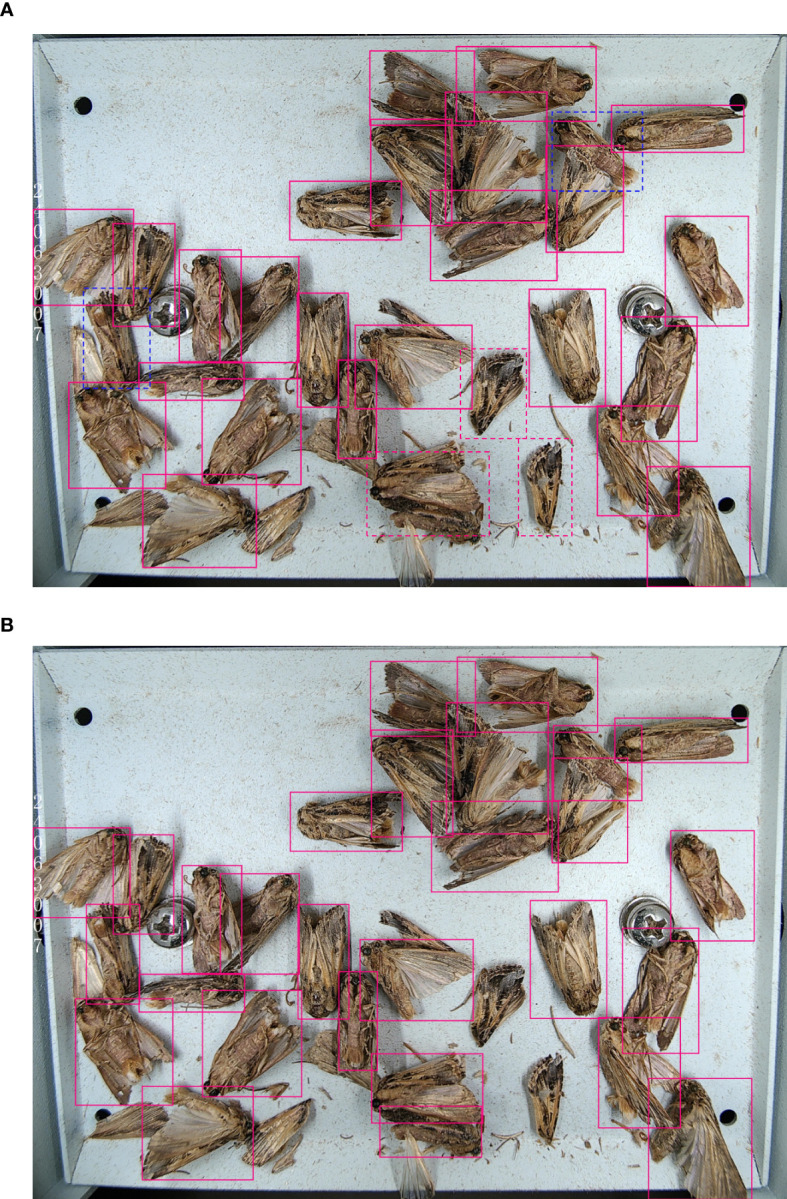
Comparison of test results. The red dashed box represents a missed detection of the *Spodoptera litura*, and the blue dashed box represents a false detection as the *Spodoptera frugiperda*: **(A)** YOLOv9 test result, **(B)** YOLOv9-TrapPest test result.

As shown in the table, original YOLOv9 model achieved a recall rate of 91.5% and a mAP50 of 93.9%. However, it exhibited several limitations, including a tendency to falsely detect separated limbs of pests as individual target pests, to incorrectly recognize multiple overlapping pests as a single target, and to misclassify visually similar pest species, leading to false detections. In Exp2, the introduction of the AKConv module resulted in a 1.9% increase in recall rate and a 1.2% increase in mAP50. AKConv addresses the issue of false detection of limb separation by adaptively adjusting the shape and parameters of the convolution kernel, better adapting the model to the morphological characteristics of various pests, and accurately capturing the entire target. In Exp3, the further addition of the CBAM-PANet module resulted in a 2.1% improvement in mAP50. This module enhances the model’s attention to important features and key regions by introducing the channel attention and spatial attention mechanisms, which makes the model perform better in processing adhesive pest images. In Exp4, the introduction of the FocalNet module resulted in a recall rate of 96.6% and a mAP50 of 98.3%. The focal modulation mechanism of FocalNet enhances the model’s ability to focus on high-confidence regions and capture fine-grained features, significantly reducing background interference and improving its capacity to distinguish between agricultural pest species with similar characteristics. As illustrated in **Figure 9**, the original YOLOv9 model faced challenges such as false detections of pest wings as target pests, missed detections due to adhered pests, and false detections when detecting visually similar pests. Yet, after incorporating the improved strategies proposed in this paper, these issues were effectively mitigated, leading to more accurate and reliable pest detection.

### 3.2 Model performance comparison experiment

To validate the detection effect of the proposed agricultural pest detection model, YOLOv6m, YOLOv8m, YOLOv9, Cascade R-CNN, and DETR were selected for the comparison experiments in this paper, and the same training set, test set, and training parameter settings were chosen for all the detection models. The detection results of different models are shown in **Table 3**.

**Table 3:** Performance comparison between YOLOv9-TrapPest and other detection models.

Models	Recall (%)	Precision (%)	mAP50 (%)
YOLOv6m	79.0	89.5	90.6
YOLOv8m	92.9	93.6	94.2
YOLOv9	91.5	92.2	94.9
Cascade R-CNN	90.7	91.7	94.4
DETR	81.1	91.3	93.8
YOLOv9-TrapPest	96.6	97.5	98.3

**Figure 10:**
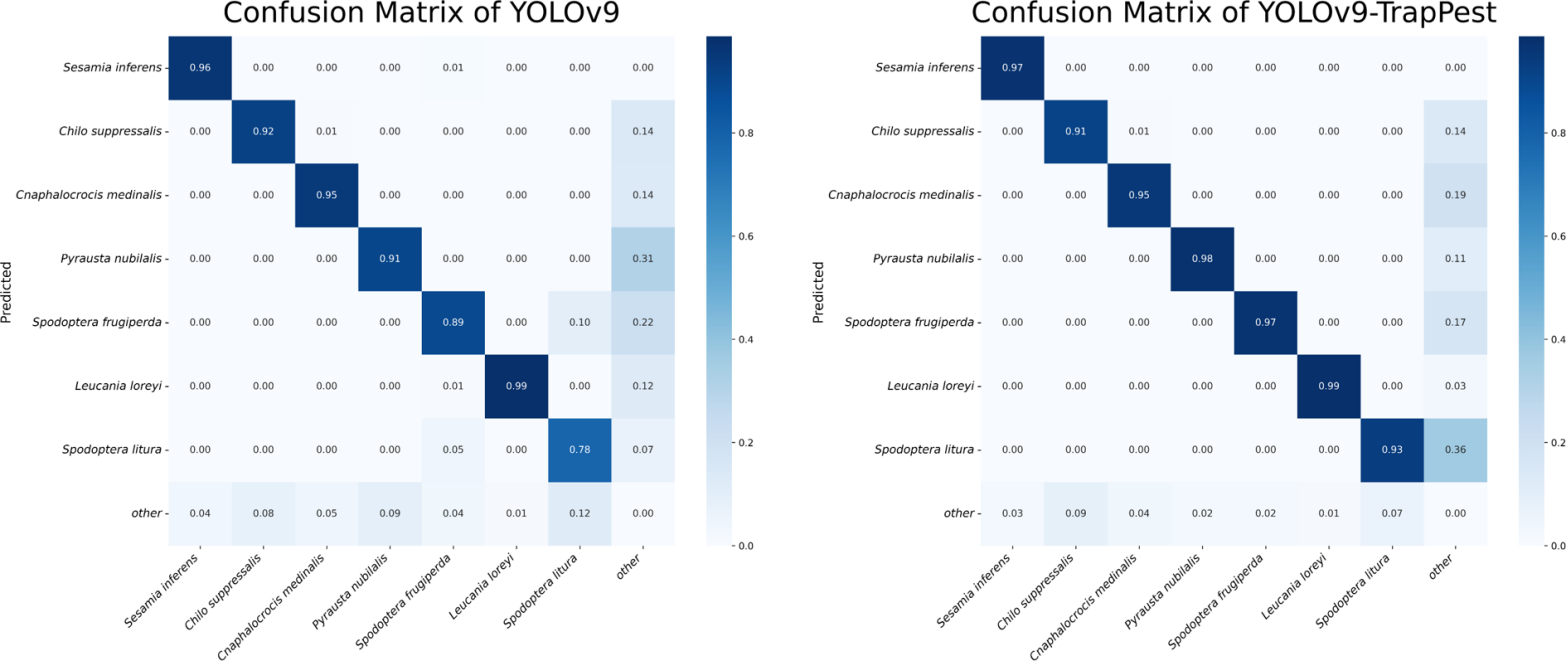
Confusion matrix of YOLOv9 and YOLOv9-TrapPest.

According to the results, the YOLOv9-TrapPest obtained the highest mAP50, which was 7.7%, 4.1%, 3.4%, 3.9%, and 4.5% higher compared to YOLOv6m, YOLOv8m, YOLOv9, Cascade R-CNN and DETR, respectively. The results of the confusion matrix for the YOLOv9-TrapPest model are shown in **Figure 10**. Due to the distinctive characteristics of the pest wings, some wings that were separated by the electric shock module were still falsely detected as target pests.

### 3.3 Interfaces of pest pheromone monitoring platform

**Figure 11:**
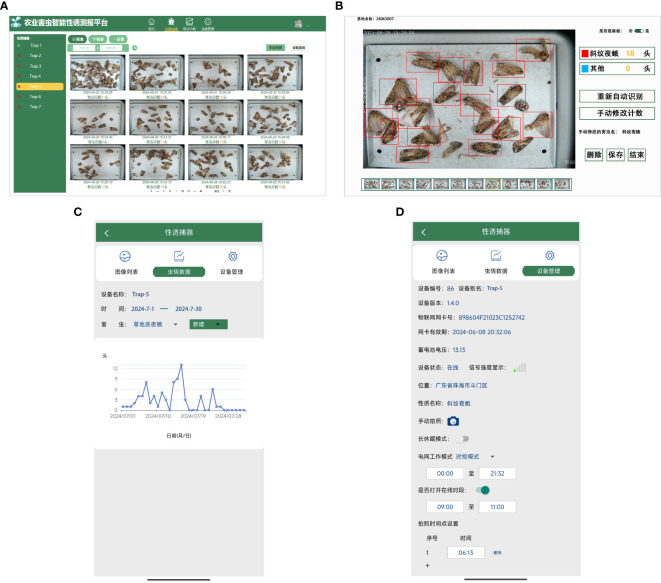
Display of the interface of the pest pheromone monitoring platform: **(A)** Image list interface in Web, **(B)** Recognition result interface in Web, **(C)** Data analysis interface in App, **(D)** Device management interface in App.

The interface of the proposed pest pheromone monitoring platform for agricultural pests is shown in **Figure 11**. It includes the image list interface, recognition result interface, data analysis interface and device management interface. In the image list interface, the images of pests acquired by smart electro-killing pheromone traps are displayed. The recognition result interface shows the results of these images processed by the algorithm, including detailed information such as the time of taking pictures, the type of pictures, and the number of various target pests. The data analysis interface statistically analyses the collected data, showing the occurrence of each type of target pest in a specific period, providing a scientific basis for agricultural pest control. The device management interface contains detailed information about the smart electro-killing pheromone traps, such as the serial number of the device, the latitude and longitude information of the device, and the power information of the device. The interface also has a remote-control function, through which users can remotely control the device to take pictures manually, thus improving the convenience and flexibility of device operation.

## 4 Discussion

As a critical monitoring tool, sex pheromone-baited technology plays an essential role in the prevention and control of agricultural pests. By employing synthetic pheromone lures to attract and trap adult pests, this technology enables precise monitoring of pest population dynamics, providing a reliable basis for developing scientifically sound pest control strategies. This method is not only efficient, environmentally friendly, and cost-effective, but it also significantly reduces the reliance on chemical pesticides, thereby lowering environmental pollution and the risk of pesticide residues on crops. Ultimately, this approach contributes to truly sustainable and green pest prevention and control practices. There are a number of commercially available sex pheromone-baited traps in the international market, such as Trapview, iSCOUT and SightTrap (Preti et al., 2021; Suto, 2022). Nonetheless, fully automated monitoring of agricultural pests remains unattainable, primarily due to the necessity of manually replacing sticky boards once a certain number of pests have adhered to them. Moreover, Schrader et al. (2022) noted that commercial remote monitoring traps are adopted hesitantly by growers due to their high costs (approximately USD 1375/ha). To solve the aforementioned problems and realize an unattended and cheaper intelligent pest monitoring system, a smart electrical pheromone trap was designed in this paper. The trap utilizes an electric shock module in place of the traditional sticky board for pest elimination, allowing for continuous dynamic updating of the photographed area. This is achieved by rotating the flat bottom tray, ensuring that the area for pest monitoring remains unobstructed and up-to-date.

Despite advancements in sex pheromone-baited traps, field placement results in pest images with complex backgrounds. This necessitates the development of robust and highly generalized pest recognition models. Miao and Zhou (2019) enhanced the SSD model to detect six types of grain storage pests, addressing challenges such as complex backgrounds, small pest sizes, and varied postures in sticky board images. Their improvements included refining the object bounding box regression strategy and loss function, optimizing the feature extraction network, and incorporating scratch-window sampling alongside multi-scale feature fusion. These enhancements led to a mAP of 81.36% for the model. Li et al. (2021) used a modified Faster-RCNN to detect whitefly and thrips on sticky traps in the greenhouse. To address the problem of poor results in detecting small targets, the scale of the anchor frames was adjusted for better coverage of small pests and RoIAlign was used instead of RoIPooling to improve the positioning accuracy of the candidate frames, with a mAP of 95.2%. Zhang et al. (2022) established a two-layer network model based on improved YOLOv3 and DBTNet-101 to detect the Cnaphalocrocis medinalis. To address the challenges of complex backgrounds, sticky pests, and reduced recognition rates caused by false detections of similar pests, the authors introduced several improvements. They incorporated DropBlock regularization to mitigate the false detection of non-targets and replaced the traditional NMS with DIoU-NMS, which effectively minimized the loss of prediction frames for sticky targets. Further, by cascading a layer of the deep bilinear transform classification network, DBTNet-101, they reduced the mutual misdetection between target and interfering pests. As a result of these enhancements, the recognition precision and recall rates for Cnaphalocrocis medinalis reached 97.6% and 98.6%, respectively.

In this paper, to address the issue of reduced model recognition accuracy caused by localized and broken pests due to the electric shock module, the AKConv module was introduced to enhance the backbone network and minimize interference from pest limbs. To tackle the problem of pests sticking together during peak infestation periods, which reduced the model’s recall rate, a CBAM-PANet structure based on the attention mechanism was proposed. This structure enables the model to more accurately identify and locate targets within complex backgrounds and images containing sticky pests. To further address the issue of false detection of similar pest species, FocalNet was incorporated in place of the SPPELAN module in the Head network. This modification allows the model to capture fine-grained features, improving its ability to differentiate between visually similar pest species.

The electro-killing pheromone-baited intelligent agricultural pest monitoring system is fully unattended and can be applied in any location where pheromone-based pest monitoring is required. This includes diverse agricultural settings such as greenhouses, orchards, and rice paddies. And its pest recognition model can be further trained to detect more pest species. Moreover, compared to pheromone-based monitoring systems utilizing machine vision, this system is more cost-effective. By integrating an electric shock module, it eliminates the need for sticky boards, thereby reducing material costs and minimizing environmental pollution caused by the sticky boards. Additionally, it reduces labor costs by eliminating the need to replace sticky boards, making it a more budget-friendly choice for growers.

## 5 Conclusions

In this paper, an electro-killing pheromone-baited intelligent agricultural pest monitoring system based on machine vision was designed and implemented. It consists of smart electro-killing pheromone traps, a server for data access and model deployment and a pest pheromone monitoring platform. Using an electric shock module and base plate flipping technology, the smart electro-killing pheromone traps efficiently electrocute pests and capture images. To address the issues of false detections caused by pest limbs after electrocution, as well as the false detection of similar pests and missed detections due to large quantities of insects sticking together during peak periods, the AKConv module, CBAM-PANet structure, and FocalNet module were integrated into the YOLOv9 model. These enhancements significantly improved the identification of seven agricultural pests, achieving an average precision rate of 97.5%, an average recall rate of 96.6%, and a mAP50 of 98.3%. The electro-killing pheromone-baited intelligent agricultural pest monitoring system eliminates the need for regular replacement of sticky boards, while providing real-time data on the number of pests trapped each day, thereby offering crucial support for informed pest control decisions.

## Data Availability

The raw data supporting the conclusions of this article will be made available by the authors, without undue reservation.
